# Use of Inverse Method to Determine Thermophysical Properties of Minimally Processed Carrots during Chilling under Natural Convection

**DOI:** 10.3390/foods12102084

**Published:** 2023-05-22

**Authors:** Wilton Pereira da Silva, Leidjane Matos de Souto, João Paulo de Lima Ferreira, Josivanda Palmeira Gomes, Antonio Gilson Barbosa de Lima, Alexandre José de Melo Queiroz, Rossana Maria Feitosa de Figueirêdo, Dyego da Costa Santos, Maristela de Fátima Simplicio de Santana, Francislaine Suelia dos Santos, Lumara Tatiely Santos Amadeu, Plúvia Oliveira Galdino, Caciana Cavalcanti Costa, Aluízio Freire da Silva Júnior, Célia Maria Rufino Franco

**Affiliations:** 1 Center for Technology and Natural Resources, Academic Unit of Agricultural Engineering, Federal University of Campina Grande, Campina Grande 58429-900, Brazil; leidjane.matos@professor.ufcg.edu.br (L.M.d.S.); joaop_l@hotmail.com (J.P.d.L.F.); josivanda@gmail.com (J.P.G.); antonio.gilson@ufcg.edu.br (A.G.B.d.L.); alexandrejmq@gmail.com (A.J.d.M.Q.); rossanamff@gmail.com (R.M.F.d.F.); francislainesuelis@gmail.com (F.S.d.S.); lumaratatielyea@gmail.com (L.T.S.A.); pluvia.oliveira@professor.ufcg.edu.br (P.O.G.); caciana.cavalcanti@professor.ufcg.edu.br (C.C.C.); aluiziofsj.ces@gmail.com (A.F.d.S.J.); celia.maria@professor.ufcg.edu.br (C.M.R.F.); 2 Federal Institute of Education, Science and Technology of Rio Grande do Norte, Paus dos Ferros 59900-000, Brazil; dyego.csantos@gmail.com; 3 National Semiarid Institute, Campina Grande 58434-700, Brazil; maristelasantana@gmail.com

**Keywords:** minimal processing, analytical solution, thermal diffusivity, heat transfer coefficient, optimization

## Abstract

The aim of this study was to determine the thermophysical properties and process parameters of cylindrical carrot pieces during their chilling. For this, the temperature of the central point of the product, initially at 19.9 °C, was recorded during chilling under natural convection, with the refrigerator air temperature maintained at 3.5 °C. A solver was created for the two-dimensional analytical solution of the heat conduction equation in cylindrical coordinates. This solver and the experimental data set were coupled to the LS Optimizer (V. 7.2) optimization software to simultaneously determine not only the values of thermal diffusivity (α) and heat transfer coefficient ($h_{H}$), but also the uncertainties of these values. These values were consistent with those reported in the literature for carrots; in this study, the precision of these values and the confidence level of the results (95.4%) were also presented. Furthermore, the Biot numbers were greater than 0.1 and less than 40, indicating that the mathematical model presented in this study can be used to simultaneously estimate *α* and $h_{H}$. A simulation of the chilling kinetics using the values obtained for *α* and $h_{H}$ showed good agreement with the experimental results, with a root mean square error RMSE = 9.651 × 10^−3^ and a chi-square χ^2^ = 4.378 × 10^−3^.

## 1. Introduction

The world production of carrot (*Daucus carota* L.) was about 42 million tons in 2021, concentrated mainly in China (18.2 million tons; 43.3% of world production) and, to a lesser extent, in Russia (1.3 million tons; 3.1% of world production) [1]. In a review study [2], the authors found that the sensory and chemical quality attributes of carrots can be influenced by several factors, such as genetic and climatic factors, cultivation method, storage temperature, and atmosphere, as well as thermal processing. Regarding the chemical composition of carrots, health-promoting bioactive compounds, such as vitamin C, phenolics (neochlorogenic and chlorogenic acid, 5-p-coumaroylquinic acid, or 3,5-dicaffeoylquinic acid), polyacetylenes (falcarinol, falcarindiol, and falcarindiol acetate), and, mainly, carotenoids (α- and β-carotene) [2,3,4,5,6,7,8], are worth mentioning. Conventionally, carrots are marketed whole, but the food industry has been dedicating a great deal of effort to developing products that combine safety, nutritional quality, and practicality in preparation. In this last aspect, minimally processed carrots are an attractive product option.

Minimal processing of fruits and/or vegetables consists of different steps, which include, but are not limited to, sanitization, removal of inedible parts, cutting or milling, packaging, and storage [9]. Despite the advantages related mainly to their easy preparation, minimally processed products (MPPs) are more perishable than their intact fresh versions due to the damage to their cellular tissue, which ends up being a window for microbial contamination and induces various deteriorating reactions [10,11,12], particularly under warmer storage conditions [13,14]. Thus, in order to extend the lifespan of MPPs, a fundamental part of the process is chilling the product and storing it under refrigeration [15,16,17,18,19]. In the chilling stage, products are typically at room temperature initially and are exposed to lower temperatures (from 2 to 10 °C) [20,21,22] until the thermal equilibrium condition is established.

Many phenomena involving food processing can be described through the diffusion equation. These phenomena include drying [23], osmotic dehydration [24,25], heating [26], and chilling [27]. To describe the chilling kinetics of a product, it is necessary to know its thermophysical properties, such as thermal diffusivity and the parameter heat transfer coefficient, among others. Thus, it is possible to estimate the minimum time for the least favorable point of the product to reach equilibrium temperature and, with this, determine the costs of the chilling process. These properties and parameters can be determined in two ways: directly and indirectly [28,29]. In the first one, direct, it is common to use specific equipment and relatively complex handling [30,31,32,33]. Conversely, the indirect way, based on the inverse method, has the advantage of being simpler and not requiring the use of any complex and/or expensive equipment. Briefly, the indirect way consists of solving, analytically or numerically, the partial differential equation of heat conduction and fitting the solution obtained, using some optimization process, to a set of experimental data of the temperature measured at a specific point of the product as a function of process time [34,35,36,37,38,39].

In a case of pasteurization, the difference between the temperature of the heated product and its initial temperature is relatively large (Δ*T* > 40 °C) [40,41,42], and thermophysical properties should generally be considered variable [43]. In the case of chilling, the difference between the initial temperature and the temperature of the cooled product is relatively small (Δ*T* < 20 °C) [27,44,45,46]. Thus, in cases of chilling, in general, thermophysical parameters can be considered constants in the description of the transient process. Thus, the analytical solution of the heat conduction equation can be used to describe the chilling kinetics of agricultural products [45,47,48]. In these cases, the properties density (*ρ*), specific heat (*C_p_*) and thermal conductivity (k) can be combined into only one, called thermal diffusivity, *α* = *k*/(*ρC_p_*) [49,50], to describe the chilling kinetics. If the boundary condition of the heat conduction equation for the process is of the first kind, this single property *α* is sufficient to describe chilling. On the other hand, if the boundary condition appropriate to the problem is of the third kind, it will also be necessary to know the heat transfer coefficient [51].

It is interesting to highlight that the literature on the determination of the thermophysical properties of carrots, processed or not, is limited. However, one example is the study conducted by Chang and Toledo [52], who used the inverse method to estimate the thermal diffusivity and heat transfer coefficient during the heating of carrot cubes. In another example, Murakami [32] indirectly estimated the thermal diffusivity of cylindrical pieces of carrots subjected to different processing techniques. However, no studies were found reporting the determination of the thermophysical properties of minimally processed carrots (MPCs) during their chilling within a given temperature range. However, accurately determining thermophysical properties is important for designing and optimizing refrigerated storage systems for MPCs. In this context, this study aimed to determine the thermophysical properties (and their uncertainties) of cylindrical pieces of carrot during their chilling using the inverse method. Such determination involved: 1—a set of experimental data of temperature at the central point of the product during the transient process; 2—a solver developed for the analytical solution of the heat conduction equation; and 3—an optimization software called LS Optimizer for Differential Equations and Functions (http://www.labfit.net/LS.htm, accessed on 1 March 2023).

## 2. Materials and Methods

### 2.1. Materials

**Figure 1:**
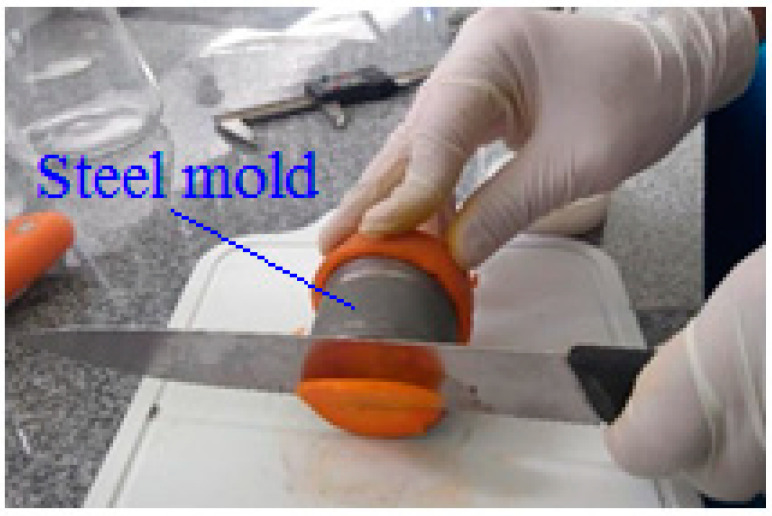
Removal of side excesses from the cylindrical piece of carrot (*D* = 44.00 mm and *L* = 40.00 mm).

Carrots were acquired at the local market in the city of Campina Grande, Paraíba, Brazil. Only specimens with similar size and without physical damage or other injuries of any nature were washed in running water to remove surface dirt. Then, they were sanitized by immersion in chlorinated solution (200 ppm) for 15 min, rinsed in running water, and then dried with absorbent paper to remove surface moisture. Finally, the carrots were peeled and cut into cylindrical pieces (diameter *D* = 44.00 mm and length *L* = 40.00 mm) with the aid of a steel mold and using a stainless steel knife (Figure 1). Diameter and length were measured using an INSIZE brand caliper, with a measuring range from 0 to 150 mm and precision of 0.01 mm.

### 2.2. Chilling Experiment

**Figure 2:**
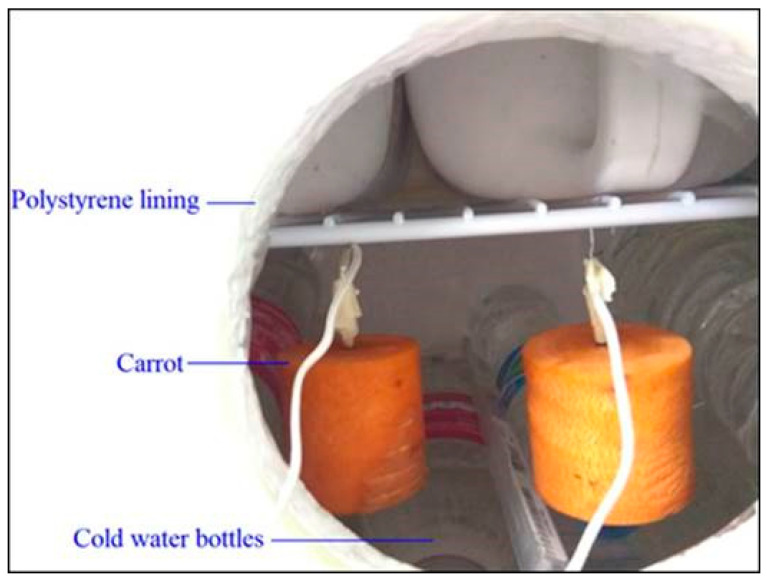
Customization of the interior of the refrigerator before the chilling experiment.

The carrot pieces, initially at 19.9 °C, were subjected to chilling in a refrigerator (Brastemp^®^) at a temperature of 3.5 °C with natural air convection. Before placing a piece of carrot in the refrigerator chamber, the temperature of the device was adjusted and maintained for 24 h. It is important to mention that, to minimize possible temperature fluctuations, the interior of the refrigerator was previously lined with polystyrene and filled with previously cooled water bottles, already at the temperature of the experiment, T = 3.5 °C (Figure 2).

Figure 2 presents an experimental setup that is useful on a laboratory scale for the determination of thermophysical properties but which obviously would not be recommended for industrial or commercial use for chilling purposes. However, the results obtained herein can be recommended, as will be observed below.

**Figure 3:**
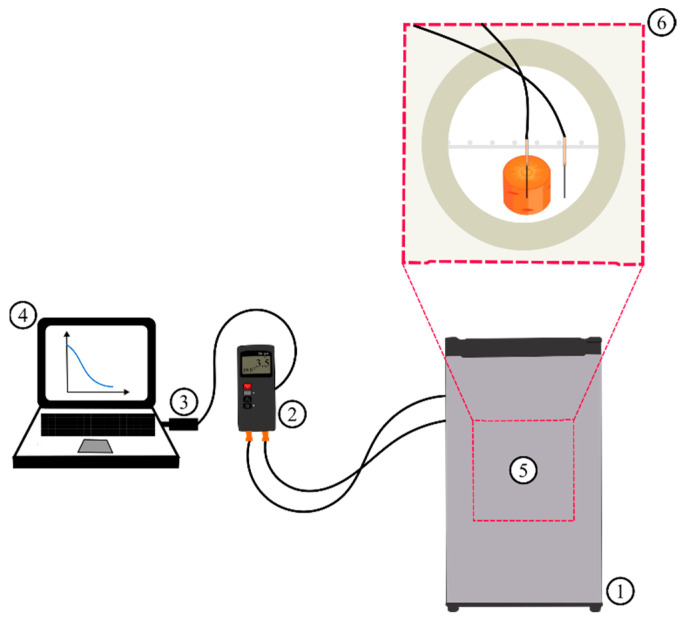
Schematic representation (without scale) of the chilling experiment configuration, with detail of the interior of the refrigerator where the carrot piece was located (without scale). (1) Refrigerator; (2) digital thermometer; (3) USB cable with serial port; (4) notebook; (5) central region of the refrigerator; and (6) detail with the representation of the interior of the central region of the refrigerator.

Automatic acquisition of temperature data at the central point of the cylindrical piece during chilling was performed using a system composed of a TH-095 Instrutherm digital thermometer, two K-type thermocouples, a USB-01 cable with an RS-232 serial port, and a Core i5 notebook. Thermocouple probes were inserted into the geometric centers of the cylindrical pieces of carrot, defined as *r* = 0 and *y* = 0, while a probe of another thermocouple was kept close to the carrot piece but in contact with the ambient air to record the temperature inside the refrigerator. Finally, the thermocouples were connected to the digital thermometer (TH-095), which sent (via USB cable) the temperature data to the notebook, which recorded these data in real time (Figure 3).

### 2.3. Governing Equation

The partial differential equation describing heat conduction in a finite cylinder with radial and axial symmetry is given by

(1) $$\rho C_{p}\frac{\partial T}{\partial t}=k\left[\frac{1}{r}\frac{\partial }{\partial r}\left(r\frac{\partial T}{\partial r}\right)+\frac{\partial^{2}T}{\partial {\;y}^{2}}\right]$$

where *t* is time (s), *T* is temperature (K), *ρ* is density (kg m^−3^), *C_p_* is specific heat (J kg^−1^ K^−1^) and *k* is thermal conductivity (W m^−1^ K^−1^). In Equation (1), *r* is the radial position defined in relation to the central axis of the finite cylinder and, together with the axial position *y* (direction of the central axis), defines the location of a point inside the cylinder. Equation (1) can also be written as

(2) $$\frac{\partial T}{\partial t}=\alpha \left[\frac{1}{r}\frac{\partial }{\partial r}\left(r\frac{\partial T}{\partial r}\right)+\frac{\partial^{2}T}{\partial {\;y}^{2}}\right]$$

where *α* is thermal diffusivity (m^2^ s^−1^) and is given by *k*/(*ρC_p_*).

To solve Equation (2) analytically, the following assumptions were made: (1) Heat generation within the product during chilling can be considered negligible; (2) initial temperature is known and uniform; (3) the geometry of the product is that of a homogeneous and isotropic finite cylinder; (4) the origin of the coordinate system is located in the geometric center of the product; (5) for the chilling temperature range, the thermophysical properties of the product can be considered constant; (6) the appropriate boundary condition for the heat conduction equation is of the third kind, and the heat transfer coefficient is the same for all product/air interfaces; and (7) the chilling temperature *T_eq_* can be considered constant throughout the process, with cold air under natural convection.

#### 2.3.1. Initial Condition and Radial and Axial Symmetry

For *t* = 0,

(3) $$T\left(r,y,0\right)\;{=T}_{0},\;\mathrm{in}\;\mathrm{the}\;\mathrm{domain}\;\left(0\;\leq \;r\;\leq \;R\;\mathrm{and}\;-\;L/2\;\leq \;y\;\leq \;L/2\right)$$

where *T*_0_ is the initial temperature (K), *R* is the cylinder radius (m), and *L* is the cylinder length (m). On the other hand, in the solution of the heat conduction equation, the assumptions of radial and axial symmetry were considered.

#### 2.3.2. Boundary Condition

The boundary condition of the third kind presupposes resistance to heat flow at the boundary of the product. Thus, the equality between the internal heat flow (diffusive) on the surface of the product and the external flow (convective) in the outer vicinity of this surface is given by Equation (4) for the radial case (*r* = *R*):

(4) $$-k{\left.\frac{\partial T\left(r,y,t\right)}{\partial r}\right|}_{r=R}=h_{H}\left[{\left.T\left(r,y,t\right)\right|}_{r=R}{\;-\;T}_{eq}\right]$$

*h_H_* is the heat transfer coefficient (W m^−2^ K^−1^).

For axial flows (*y* = ±*L*/2), the boundary condition of the third kind is given by Equation (5):

(5) $$-k{\left.\frac{\partial T\left(r,y,t\right)}{\partial y}\right|}_{y=\pm L/2}=h_{H}\left[{\left.T\left(r,y,t\right)\right|}_{y=\pm L/2}\;{-\;T}_{eq}\right]$$

where *L* is cylinder length (m) and ${\left.T\left(r,y,t\right)\right|}_{y=\pm L/2}$ is temperature (K) in the circular areas located in positions *y* = ±*L*/2.

#### 2.3.3. Analytical Solution of the Heat Conduction Equation

Considering the simplifying assumptions presented above, the solution of Equation (2) can be obtained by the separation of variables [53,54].

(6) $$T\left(r,y,t\right){=T}_{eq}+\left(T_{0}\;{-\;T}_{eq}\right)\overset{\infty }{\underset{n=1}{\sum }}\overset{\infty }{\underset{m=1}{\sum }}A_{n}A_{m}J_{0}\left(\mu_{n}\frac{r}{R}\right)\cos\left(\mu_{m}\frac{y}{L/2}\right)\;\times \;\exp\left[-\left(\frac{\mu_{n}^{2}}{R^{2}}+\frac{\mu_{m}^{2}}{{\left(L/2\right)}^{2}}\right)\alpha t\right]$$

where *T*(*r*,*y*,*t*), *T*_0_, and *T_eq_* are: (1) the temperature in a position (*r*,*y*) of the cylinder at an instant *t*; (2) the initial temperature of the product; and (3) the equilibrium temperature after chilling. In Equation (6), the parameter *α* is thermal diffusivity, and the coefficients *A_n_* and *A_m_* are calculated as follows:

(7) $$A_{n}=\frac{{2Bi}_{1}}{J_{0}\left(\mu_{n}\right)\left({Bi}_{1}^{2}{+\mu }_{n}^{2}\right)}$$

and

(8) $$A_{m}={\left(-1\right)}^{m+1}\frac{{2Bi}_{2}{\left({Bi}_{2}^{2}{+\mu }_{m}^{2}\right)}^{1/2}}{\mu_{m}\left({Bi}_{2}^{2}{+Bi}_{2}{+\mu }_{m}^{2}\right)}$$

In Equations (7) and (8), *Bi*_1_ and *Bi*_2_ are the Biot numbers for heat transfer in an infinite cylinder and on an infinite wall, respectively, and are calculated according to Equations (9) and (10), in this order:

(9) $${Bi}_{1}=\frac{h_{H}R}{k}$$

and

(10) $${Bi}_{2}=\frac{h_{H}L/2}{k}$$

In Equations (9) and (10), *h_H_* is the heat transfer coefficient, which was considered to have the same value for all cylinder surfaces. In Equations (6)–(8), *μ_n_* and *μ_m_* are the roots of the characteristic equations for an infinite cylinder and an infinite wall, respectively, and were calculated using the following equations:

(11) $$\frac{J_{0}\left(\mu_{n}\right)}{J_{1}\left(\mu_{n}\right)}=\frac{\mu_{n}}{{Bi}_{1}}$$

and

(12) $${\cot\;\mu }_{m}=\frac{\mu_{m}}{{Bi}_{2}}$$

In Equation (11), *J*_0_ and *J*_1_ are the first-kind Bessel functions of orders 0 and 1, respectively. On the other hand, Equation (6) can be defined in terms of a dimensionless temperature as follows:

(13) $$T^{*}\left(r,y,t\right)=\frac{T\left(r,y,t\right)\;{-\;T}_{eq}}{T_{0}{-T}_{eq}}=\overset{\infty }{\underset{n=1}{\sum }}\overset{\infty }{\underset{m=1}{\sum }}A_{n}A_{m}J_{0}\left(\mu_{n}\frac{r}{R}\right)\cos\left(\mu_{m}\frac{y}{L/2}\right)\;\times \;\exp\left[-\left(\frac{\mu_{n}^{2}}{R^{2}}+\frac{\mu_{m}^{2}}{{\left(L/2\right)}^{2}}\right)\alpha t\right]$$

### 2.4. Proposed Heat Transfer Model for Chilling

#### 2.4.1. Direct Problem: Solver Development

Equation (6) is used to determine the temperature *T*(*r*,*y*,*t*), whereas Equation (13) can also be used to determine the dimensionless temperature T*(*r*,*y*,*t*), for previously defined values of *h* and *α*. For this, the Biot numbers were calculated by Equations (9) and (10). The first 200 roots of Equations (11) and (12) were calculated by the bisection method, with a convergence tolerance of 10^−12^. This allowed determining the first 200 coefficients, *A_n_* and *A_m_*, given by Equations (7) and (8), respectively. Thus, for the analytical solution of the partial differential equation of heat conduction, a solver was created in FORTRAN. The developed software was created in Compaq Visual Fortran Professional Studio, V. 6.6.0, using a programming language option called QuickWin Application.

#### 2.4.2. Inverse Problem: Simultaneous Determination of Optimal Values of *α* and $h_{H}$

The LS Optimizer software, developed by the first author of this article, was used to determine the values of *α* and $h_{H}$. For this, the solver created according to the previous sections and the experimental data set of temperature at the central point of the product were coupled to the LS environment. It is worth pointing out that LS Optimizer is ready-to-use software that uses non-linear regression (least squares) to determine parameters of an ordinary or partial differential equation (and functions) through known experimental data, using the Levenberg–Marquardt algorithm [55,56]. LS runs the solver to obtain the information needed for the determination of parameters. Thus, starting from initial values *α*_0_ and *h*_0_, LS provides the optimal values for *α* and $h_{H}$, their uncertainties, and the covariance matrix. In this study, the initial values of *α* and h were established as *α*_0_ = 1 × 10^−7^ m^2^ s^−1^ and $h_{H0}$ = 4 W m^−2^ K^−1^. It is interesting to mention that the solver to be run by LS Optimizer was developed to read an “exp.txt” file, which contains the experimental data in three columns: the first with data for the independent variable (*t*), the second with values for the dependent variable ($T_{i}^{\exp})$ at the central point, and the third column with the uncertainty values of the dependent variable ($\sigma_{T_{i}}$), if known. However, if this last piece of information is not known, the user just needs to write the value equal to 1, and the LS already identifies the absence of these uncertainties. It is worth mentioning that the developed solver must read a file called “parameters.txt”, generated by LS Optimizer, with the information given below. The first piece of information is an integer variable called “information”, which can be, in this case, 0, 1, or 2. The specific value defines the name of the file to be generated by the solver, which can be unsteady.txt (if the value is 0), unsteady_a1.txt (if the value is 1), or unsteady_a2.txt (if the value is 2). Thus, after the information provided by the LS is identified, the solver stores the simulation results in the appropriate file for subsequent calculations of the sensitivities of the parameters to be determined. The second piece of information is an integer variable called “N_Param” that indicates the number of parameters involved in the solution of the differential equation (in this case, N_Param = 2). Finally, the third piece of information indicates the values established by the LS Optimizer for the parameters in each iteration (in this case, only two parameters: *a*_1_ = *α* and *a*_2_ = *h*). Thus, the solver calculates the dependent variable for the same values as the independent variable specified in the experimental dataset (“exp.txt”) and then writes the simulations obtained in the files mentioned above (unsteady.txt, unsteady_a1.txt, or unsteady_a2.txt). These files are then used by LS Optimizer to calculate the chi-square in each iteration $\left(\chi^{2}=\sum {\left(T_{i}{}^{\exp}{\;-\;T}_{i}{}^{\mathrm{sim}}\right)}^{2}/\sigma_{T_{i}}{}^{2}\right)$ and also the corrections (Δ*a*_1_ and Δ*a*_2_) of the fitting parameters (*a*_1_ and *a*_2_, respectively). With the corrections to the parameters determined, the new values of the fitting parameters a_1_ (*a*_1_ = *a*_10_ + Δ*a*_1_) and *a*_2_ (*a*_2_ = *a*_20_ + Δ*a*_2_) are calculated. These corrected values are then considered new initial values (*a*_10_ and *a*_20_), and the iterative process occurs until *a* convergence criterion ($\left|\Delta a_{1}/a_{1}\right|{\;<\;1\;\times \;10}^{-5}$ and $\left|\Delta a_{2}/a_{2}\right|{\;<\;1\;\times \;10}^{-5}$) is reached. As additional information, the LS Optimizer software is freely available for download at the link provided at the end of the introduction to this article. On the other hand, in each iteration, the corrections Δ*a*_1_ and Δa_2_ are calculated by minimizing the objective function (in the present case, chi-square, χ^2^) [44], resulting in the following system of equations given in matrix form:

(14) $$\left[M\right]\left[\Delta A\right]=\left[C\right]$$

where

(15) $$\left[M\right]=\left[\begin{array}{cc} \sum \frac{{\left.\frac{\partial T_{i}^{\mathrm{sim}}}{\partial a_{1}}\right|}_{a_{1}{=a}_{10}}{\left.\frac{\partial T_{i}^{\mathrm{sim}}}{\partial a_{1}}\right|}_{a_{1}{=a}_{10}}}{\sigma_{T_{i}}^{2}} & \sum \frac{{\left.\frac{\partial T_{i}^{\mathrm{sim}}}{\partial a_{1}}\right|}_{a_{1}{=a}_{10}}{\left.\frac{\partial T_{i}^{\mathrm{sim}}}{\partial a_{2}}\right|}_{a_{2}{=a}_{20}}}{\sigma_{T_{i}}^{2}}\\ \sum \frac{{\left.\frac{\partial T_{i}^{\mathrm{sim}}}{\partial a_{2}}\right|}_{a_{2}{=a}_{20}}{\left.\frac{\partial T_{i}^{\mathrm{sim}}}{\partial a_{1}}\right|}_{a_{1}{=a}_{10}}}{\sigma_{T_{i}}^{2}} & \sum \frac{{\left.\frac{\partial T_{i}^{\mathrm{sim}}}{\partial a_{2}}\right|}_{a_{2}{=a}_{20}}{\left.\frac{\partial T_{i}^{\mathrm{sim}}}{\partial a_{2}}\right|}_{a_{2}{=a}_{20}}}{\sigma_{T_{i}}^{2}} \end{array}\right]$$

and

(16) $$\left[\Delta A\right]=\left[\begin{array}{c} \Delta a_{1}\\ \Delta a_{2} \end{array}\right]\;\mathrm{and}\;\left[C\right]=\left[\begin{array}{c} \sum \frac{{\delta T}_{i}{\left.\frac{\partial T_{i}^{\mathrm{sim}}}{\partial a_{1}}\right|}_{a_{1}{=a}_{10}}}{\sigma_{T_{i}}^{2}}\\ \sum \frac{{\delta T}_{i}{\left.\frac{\partial T_{i}^{\mathrm{sim}}}{\partial a_{2}}\right|}_{a_{2}{=a}_{20}}}{\sigma_{T_{i}}^{2}} \end{array}\right]$$

The sums range from *i* equal to 1 to *N*, which is the number of experimental points. Additionally,

(17) $${\delta T}_{i}{=T}_{i}^{\exp}\;-\;{\left.T_{i}^{\mathrm{sim}}\right|}_{\begin{array}{c} a_{1}{=a}_{10}\\ a_{2}{=a}_{20} \end{array}}$$

where “*i*” is the *i*th experimental point. In the last equations, $T_{i}^{\exp}$ are the experimental values of the temperature and ${\left.T_{i}^{\mathrm{sim}}\right|}_{\begin{array}{c} a_{1}=a_{10}\\ a_{2}=a_{20} \end{array}}$ are simulated values obtained by the developed solver by substituting *a*_1_ and *a*_2_ by *a*_10_ and *a*_20_, respectively. For more details on the mathematical foundations of LS Optimizer software, the studies conducted by Pereira et al. [57] and Silva et al. [43] can be consulted. As additional information, when the relative tolerance on the two parameters is reached in the iterative process, the inverse of Equation (15) is the covariance matrix.

## 3. Results

**Figure 4:**
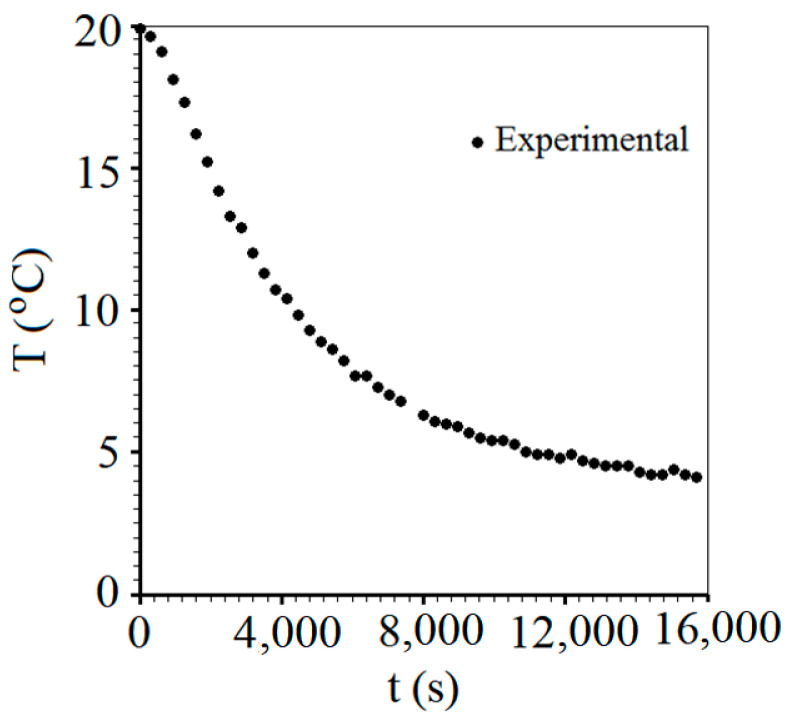
Acquisition of data of temperature *T* (°C) versus time *t* (s) at the central point of the carrot piece during chilling under natural convection.

Several tests were carried out with different pieces of carrot, whose results agreed with each other. However, we have chosen only one dataset for the analyses: the one with the least noise during data acquisition. Thus, the acquisition of temperature data at the central point of the cylindrical piece of carrot under natural convection of cold air resulted in the graph shown in Figure 4, which contains 49 experimental points.

At this point, it is important to emphasize that the values of the uncertainties of the properties and parameters, objects of this study, are not determined by repetitions of the measurement process (replicates), but through the covariance matrix, [M]^−1^. This matrix is the inverse of the last matrix [M] calculated by Equation (15) in the iterative process, that is, the one obtained in the convergence of the iterative process referring to the non-linear regression.

The temperature data throughout the transient regime were then written in the dimensionless form, as explained by Equation (13), $T^{*}\left(r,y,t\right)=(T\left(r,y,t\right)\;{-\;T}_{\mathrm{eq}}{)/(T}_{0}\;{-\;T}_{\mathrm{eq}})$. Thus, the results obtained according to the proposed methodology were presented and analyzed as shown below.

### 3.1. Thermophysical Properties and Parameters

For the chilling experiment, the initial temperature of the cylindrical piece of carrot was 19.9 °C, and the chilling temperature of the refrigerator chamber was maintained at approximately 3.5 °C. The cylindrical piece of carrot had a mass of 61.0 g and a moisture content on a wet basis of *X_wb_* = 90.4%. Its radius was *R* = 0.022 m, and its length was *L* = 0.040 m. Thus, the density could be calculated directly from the equation *ρ* = m/V, resulting in *ρ* = 1003 kg m^−3^. The specific heat of the product was estimated by the model of Fikiin [58], *C_p_* = 1382 + 2805 *X_wb_*/100, and the result was *C_p_* = 3918 J kg^−1^ K^−1^. Table 1 shows *α* and *h*, determined by optimization using: (1) the experimental data set of dimensionless temperatures at the center of the product during its chilling; (2) the solver developed to predict these dimensionless temperatures for *α* and *h* stipulated by the LS Optimizer; and (3) the LS Optimizer optimization software. The values obtained for *α* and h were multiplied by *ρC_p_* to obtain the thermal conductivity, *k*, and the heat transfer coefficient, *h_H_*, respectively. Such results are also presented in Table 1. The result obtained through the proposed model for the thermal diffusivity value of carrot was *α* = (1.43 ± 0.18) × 10^−7^ m^2^ s^−1^, with a confidence level of 95.4%. This result was consistent with the value obtained from the estimate by Riedel correlation (*α* = 0.88 × 10^−7^ + 0.6 × 10^−7^ × *X_wb_*/100) [59], *α* = 1.42 × 10^−7^ m^2^ s^−1^, and also with the value that Murakami [32] reported for fresh carrots (*X_wb_* = 87.6%; 25 °C): *α* = (1.44 ± 0.01) × 10^−7^ m^2^ s^−1^. Chang and Toledo [52] determined the thermal diffusivity, *α* = (1.86 ± 0.04) × 10^−7^ m^2^ s^−1^, of carrot pieces with a moisture content of *X_wb_* = 89.2%, at 138 °C. However, this estimate, at such a high temperature, could not really have good agreement with the results obtained in the present study, whose temperature range was 19.9 to 3.5 °C. On the other hand, the thermal conductivity value obtained using the sweat correlation [60] (*k* = 0.148 + 0.493 *X_wb_*/100) was *k* = 0.594 W m^−1^ K^−1^. This value is compatible with that obtained in the present study (*k* = *αρC_p_*), which was *k* = (0.56 ± 0.07) W m^−1^ K^−1^, with a confidence level of 95.4%.

It is interesting to point out that, although Murakami [32] determined the uncertainty of the *α* value by error propagation (considering that the properties *k*, *ρ*, and *C_p_* are determined independently of each other, that is, covariance equal to zero between each pair of values), the confidence level was not specified. In the case of the study conducted by Chang and Toledo [52], the uncertainty of the result obtained for *α* was determined using 10 repetitions, which implies a confidence level of less than 68%. On the other hand, in the present study, it was possible to determine the values of *α* and *h* (and their uncertainties) using 49 experimental points obtained for the dimensionless temperature of the center point of the cylindrical piece during the transient process. In addition, the uncertainties originally provided by the LS Optimizer program for *α* and h were multiplied by the factor 2, which was recommended by the optimization software to ensure the 95.4% confidence level (assuming a Gaussian distribution of errors). It should also be noted that an additional advantage of the proposed model is the possibility of determining the values of Biot numbers for the infinite cylinder (*Bi*_1_) and infinite wall (*Bi*_2_) (Table 1), referring to the chilling of carrot pieces with the geometry of a finite cylinder. The low values of Biot numbers (*Bi*_1_ = 0.271 and *Bi*_2_ = 0.246), presented in Table 1, indicate the importance of considering the external resistance to heat transfer between the product and the cold air around its vicinities. Thus, it can be inferred that the boundary condition of the third kind, used in this study, is actually the appropriate boundary condition to describe the chilling process of cylindrical pieces of carrot under the natural convection of cold air.

The covariance matrix provided by the LS program is given by Equation (18).

(18) $${\left[M\right]}^{-1}=\left[\begin{array}{cc} 8{.2578\;\times \;10}^{-17}\; & -1{.8611\;\times \;10}^{-16}\\ -1{.8611\;\times \;10}^{-16} & 5{.2435\;\times \;10}^{-16} \end{array}\right]$$

The covariance matrix, given by Equation (18), indicates that the parameters *α* and h are dependent on each other, and this dependence results in a correlation equal to −0.8944. This means that when the value of one of the parameters tends to increase, the value of the other parameter tends to decrease, which is common in optimization procedures involving two parameters. In this case, in error propagation calculations involving *α* and h, the covariance between these parameters must necessarily be considered. Similar results for correlation were found in several studies involving the determination of two parameters that describe phenomena involving the processing of food products [23,45,61,62].

### 3.2. Product Chilling Kinetics

**Figure 5:**
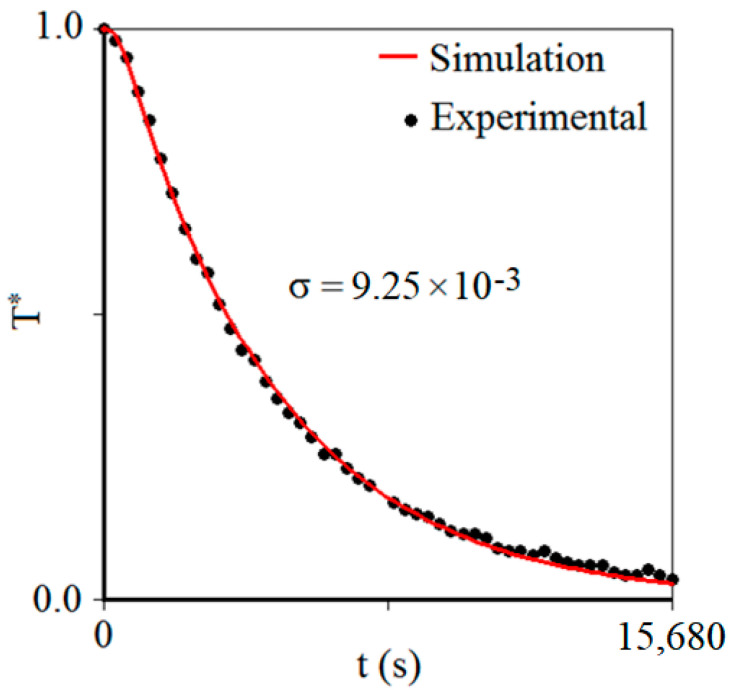
Chilling kinetics of the carrot piece simulated using the *α* and *h* values determined by optimization. The statistical indicator *σ* is the standard deviation of the fit.

With the results obtained for *α* and *h*, it was possible to simulate the chilling kinetics for the central point of the carrot piece, as shown in Figure 5.

**Figure 6:**
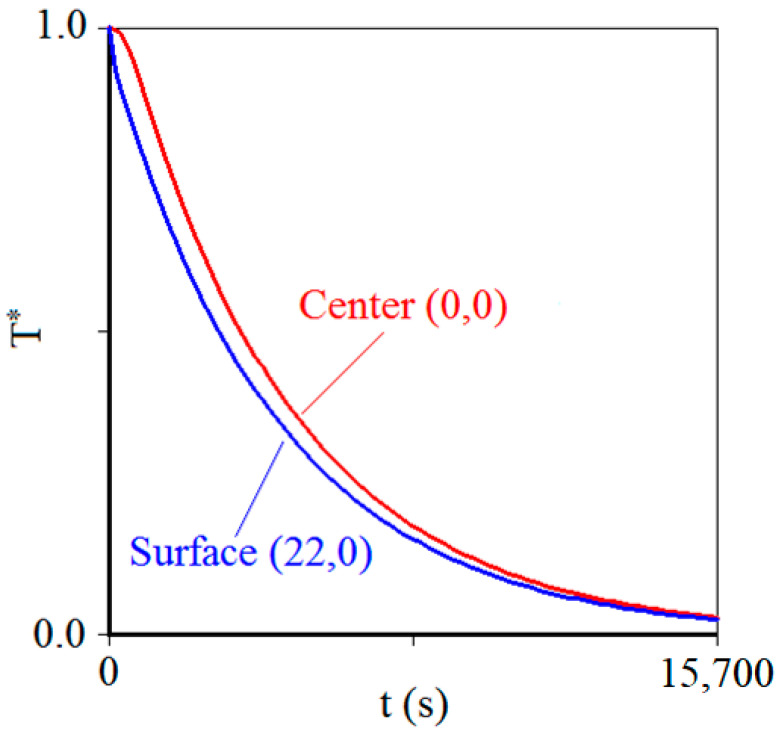
Simulations of the chilling kinetics of the carrot piece using the values of *α* and *h* determined by optimization for the center point (0,0), red line, and for the surface of the carrot piece (22,0), blue line.

A visual inspection of Figure 5 shows that the fit of the simulated line to the experimental data can be considered good. This qualitative observation is also verified by the high value of the determination coefficient (*R*^2^ = 0.9991), the low values of chi-square (*χ*^2^ = 4.378 × 10^−3^), root mean square error (RMSE = 9.651 × 10^−3^), and the standard deviation of the fit (*σ* = 9.25 × 10^−3^). On the other hand, the determination of the values for *α* and $h_{H}$ allows simulating the chilling kinetics not only in the center of the carrot piece, at coordinates (0,0), but also at any other point, such as at the boundary of the product, at the coordinate point (22,0). A graph that shows these two simulations is presented in Figure 6. This figure shows what is expected: in intermediate times of chilling kinetics, the dimensionless temperature of the center is greater than the dimensionless temperature on the surface of the product, and this difference was maximum (ΔT* = 0.11) at the instant *t* = 633.6 s (that is, *t* = 10.56 min).

It is interesting to highlight that, although the results observed in Figure 6 are expected, the proposed model can predict in detail such results for this transient process. It is also interesting to observe in Figure 6 that the temperature on the surfaces of the cylinder decreases slowly, a boundary condition of the third kind for the description of carrot chilling under natural convection. Thus, low Biot numbers could have been expected, as indeed they were.

**Figure 7:**
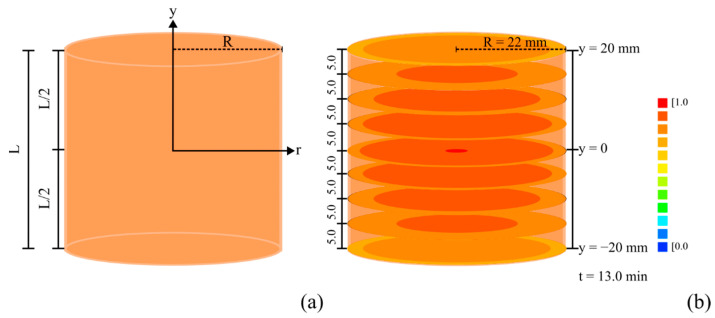
(**a**) Representation of a carrot piece as a finite cylinder with radius *R* = 22.00 mm and length *L* = 40.00 mm; (**b**) Dimensionless temperature distribution obtained using the optimized values of *α* and *h* at the instant *t* = 780 s (13.0 min) for circular areas located between *y* = −20.00 mm and *y* = 20.00 mm.

The solver developed for the proposed model also allowed predicting temperature distributions in a circle at a given axial position *y* within the finite cylinder representing the carrot piece, which is shown in Figure 7a. Such distributions can be seen in Figure 7b for circles in different positions between *y* = −20.00 mm and *y* = +20.00 mm at time *t* = 13 min. For *y* = 0, for example, it is possible to observe that the chilling front advances from the outer surface toward the center of the product.

This type of temperature distribution has also been described in studies on the chilling of different agricultural products, such as fig [27], cucumber [63], banana [45], potato [64], and apple [46]. Although this is a fairly obvious phenomenon, the proposed model makes it possible to predict these dimensionless temperature distributions at any given instant, as can be seen, for example, for the circular area in *y* = 0, during the first 13.0 min (780 s) of chilling, through the following link: http://labfit.net/Cooling.gif (accessed on 1 March 2023). Once again, it is evident from Figure 7b that, indeed, the boundary condition appropriate for the description of carrot chilling under natural convection is the third kind. Additionally, the animation shown via the link above clearly indicates that there are temperature gradients inside the carrot piece, with the temperature at the boundary varying over time.

According to many authors, such as Brischetto [65], if *Bi* < 0.1, a lumped model can be used to describe heat transfer problems with these Biot numbers, and the error associated with using this model to describe such problems is small. In this case, only the heat transfer coefficient can be determined. On the other hand, if *Bi* > 40, which corresponds practically to an infinite value, the appropriate boundary condition is of the first kind (and only the property of thermal diffusivity can be determined).

Unlike natural convection, it is interesting to observe that, depending mainly on the velocity of the coolant fluid, the heat transfer can be better described by the diffusion equation with the first kind of boundary condition. In this case, the equations describing the chilling process are the same, Equation (6) or (13), but the coefficients given by Equations (7) and (8) are defined as [44]:

(19) $$A_{n}=\frac{2}{\mu_{n}J_{1}\left(\mu_{n}\right)}$$

and

(20) $$A_{m}={\left(-1\right)}^{m+1}\frac{2}{\mu_{m}}$$

Additionally, when *Bi*_1_ and *Bi*_2_ tend to be infinite (in practical terms, *Bi* > 40), Equations (11) and (12) become $J_{0}\left(\mu_{n}\right)=0$ and ${\cot\;\mu }_{m}=0$, respectively.

## 4. Conclusions

As *Bi*_1_ and *Bi*_2_ are greater than 0.1 and both values are less than 40, it can be concluded that the mathematical modeling presented in this article is adequate to describe the chilling of the carrot pieces under natural convection. On the other hand, the thermophysical properties of cylindrical carrot pieces were determined using non-linear regression during a transient process. The result obtained for the thermal diffusivity value of the product was *α* = 1.43 × 10^−7^ m^2^ s^−1^, using the proposed model: (1) Data set of temperature in the center of the product; (2) solver developed; (3) LS Optimizer software. This result is consistent with the values found in the literature for similar temperatures of the product. However, the advantage of the proposed model is that it provides the temperature range within which this value is valid (from 19.9 down to 3.5 °C) and, mainly, its uncertainty with a confidence level of 95.4%. From the results obtained for the heat transfer coefficient (*h_H_* = 6.92 W m^−2^ K^−1^) and also for the Biot numbers (*Bi*_1_ = 0.272 and *Bi*_2_ = 0.246), it can be concluded that the surface resistance to heat flow should be considered in the description of the chilling process of carrot pieces with natural convection of cold air. Thus, the proposed model was adequate to describe the chilling of carrot pieces with the geometry of a finite cylinder, and the boundary condition appropriate to the process is of the third kind.

## Figures and Tables

**Table 1:** Thermophysical properties and parameters determined for a carrot piece (*X_wb_* = 90.4%) during chilling from 19.9 °C down to 3.5 °C. The intervals are given with a 95.4% confidence level.

Properties	Carrot	Reference
*α* (m^2^ s^−1^)	(1.43 ± 0.18) × 10^−7^	Proposed model
*k* (W m^−1^ K^−1^)	0.56 ± 0.07	Proposed model
*C_p_* (J kg^−1^ K^−1^)	3918	Fikiin [58]
*ρ* (kg m^−3^)	1003	Proposed model
**Parameters**		
*h_H_* (W m^−2^ K^−1^)	6.92 ± 0.20	Proposed model
*Bi*_1_ (−)	0.271	Proposed model
*Bi*_2_ (−)	0.246	Proposed model

## Data Availability

The data presented in this study are available upon request from the corresponding authors.

## Nomenclature

*A_n_*; *A_m_*	Coefficients of the analytical solution
*a*_1_; *a*_2_	Fitting parameters
*a*_10_; *a*_20_	Initial values of the fitting parameters
*Bi*_1_; *Bi*_2_	Biot numbers for heat transfer (dimensionless)
*C_p_*	Specific heat (J kg^−1^ K^−1^)
*h_H_*	Heat transfer coefficient (W m^−2^ K^−1^)
*J*	Bessel function
*k*	Thermal conductivity (W m^−1^ K^−1^)
*L*	Height of the cylinder (m)
M^−1^	Covariance matrix
*R*	Radius of the finite cylinder (m)
*R* ^2^	Determination coefficient (dimensionless)
*r*	Radial position in cylindrical coordinates (m)
*t*	Time (s)
*T*	Temperature (K)
*T_eq_*	Equilibrium temperature (K)
*T*_0_	Initial temperature (K)
*T* ^sim^	Temperature simulated by the solver (K)
*T* ^exp^	Experimental value of temperature (K)
*y*	Axial position in cylindrical coordinates (m)
**Greek symbols**	
*α*	Thermal diffusivity (m^2^ s^−1^)
Δ*a*_1_; Δ*a*_2_	Corrections of the fitting parameters
*χ* ^2^	Chi-square or objective function
*μ_n_*, *μ_m_*	Roots of the characteristic equations
*ρ*	Density (kg m^−3^)
*σ_T_*	Uncertainty of experimental temperature (K)

## References

[1] FAOSTAT Statistics Division, Food and Agriculture Organization of the United Nations. https://www.fao.org/faostat/en/#data.

[2] Alasalvar C., Grigor J.M., Zhang D., Quantick P.C., Shahidi F. (2001). Comparison of Volatiles, Phenolics, Sugars, Antioxidant Vitamins, and Sensory Quality of Different Colored Carrot Varieties. J. Agric. Food Chem..

[3] Czepa A., Hofmann T. (2004). Quantitative Studies and Sensory Analyses on the Influence of Cultivar, Spatial Tissue Distribution, and Industrial Processing on the Bitter Off-Taste of Carrots (*Daucus carota* L.) and Carrot Products. J. Agric. Food Chem..

[4] Ma T., Tian C., Luo J., Zhou R., Sun X., Ma J. (2013). Influence of technical processing units on polyphenols and antioxidant capacity of carrot (*Daucus carrot* L.) juice. Food Chem..

[5] Ahmad T., Cawood M., Iqbal Q., Ariño A., Batool A., Tariq R.M.S., Azam M., Akhtar S. (2019). Phytochemicals in *Daucus carota* and Their Health Benefits—Review Article. Foods.

[6] Skoczylas Ł., Tabaszewska M., Smoleń S., Słupski J., Liszka-Skoczylas M., Barański R. (2020). Carrots (*Daucus carota* L.) Biofortified with Iodine and Selenium as a Raw Material for the Production of Juice with Additional Nutritional Functions. Agronomy.

[7] Hammaz F., Charles F., Kopec R.E., Halimi C., Fgaier S., Aarrouf J., Urban L., Borel P. (2021). Temperature and storage time increase provitamin A carotenoid concentrations and bioaccessibility in post-harvest carrots. Food Chem..

[8] Aubert C., Bruaut M., Chalot G. (2022). Spatial distribution of sugars, organic acids, vitamin C, carotenoids, tocopherols, 6-methoxymellein, polyacetylenic compounds, polyphenols and terpenes in two orange Nantes type carrots (*Daucus carota* L.). J. Food Compos. Anal..

[9] Testa R., Schifani G., Migliore G. (2021). Understanding Consumers’ Convenience Orientation. An Exploratory Study of Fresh-Cut Fruit in Italy. Sustainability.

[10] Ali S., Khan A.S., Anjum M.A., Nawaz A., Naz S., Ejaz S., Hussain S. (2020). Effect of postharvest oxalic acid application on enzymatic browning and quality of lotus (*Nelumbo nucifera* Gaertn.) root slices. Food Chem..

[11] Wang D., Li W., Li D., Li L., Luo Z. (2021). Effect of high carbon dioxide treatment on reactive oxygen species accumulation and antioxidant capacity in fresh-cut pear fruit during storage. Sci. Hortic..

[12] Chen J., Xu Y., Yi Y., Hou W., Wang L., Ai Y., Wang H., Min T. (2022). Regulations and mechanisms of 1-methylcyclopropene treatment on browning and quality of fresh-cut lotus (*Nelumbo nucifera* Gaertn.) root slices. Postharvest Biol. Technol..

[13] Poubol J., Izumi H. (2005). Shelf Life and Microbial Quality of Fresh-cut Mango Cubes Stored in High CO_2_ Atmospheres. J. Food Sci..

[14] Min T., Xie J., Zheng M., Yi Y., Hou W., Wang L., Ai Y., Wang H. (2017). The effect of different temperatures on browning incidence and phenol compound metabolism in fresh-cut lotus (*Nelumbo nucifera* G.) root. Postharvest Biol. Technol..

[15] Allong R., Wickham L., Mohammed M. (2000). The effect of cultivar, fruit ripeness, storage temperature and duration on quality of fresh-cut mango. Acta Hortic..

[16] Rattanapanone N., Lee Y., Wu T., Watada A.E. (2001). Quality and Microbial Changes of Fresh-cut Mango Cubes Held in Controlled Atmosphere. Hortscience.

[17] Izumi H., Nagatoma T., Tanaka C., Kanlayanarat S. (2003). Physiology and quality of fresh-cut mango is affected by low O_2_ controlled atmosphere storage, maturity and storage temperature. Acta Hortic..

[18] Poubol J., Izumi H. (2006). Physiology and Microbiological Quality of Fresh-cut Mango Cubes as Affected by High-O_2_ Controlled Atmospheres. J. Food Sci..

[19] Liu X., Wang T., Lu Y., Yang Q., Li Y., Deng X., Liu Y., Du X., Qiao L., Zheng J. (2019). Effect of high oxygen pretreatment of whole tuber on anti-browning of fresh-cut potato slices during storage. Food Chem..

[20] Kakiomenou K., Tassou C., Nychas G. (1996). Microbiological, physicochemical and organoleptic changes of shredded carrots stored under modified storage. Int. J. Food Sci. Technol..

[21] Wang C., Chen Y., Xu Y., Wu J., Xiao G., Zhang Y., Liu Z. (2013). Effect of dimethyl dicarbonate as disinfectant on the quality of fresh-cut carrot (*Daucus carota* L.). J. Food Process. Preserv..

[22] Kapusta-Duch J., Kusznierewicz B., Leszczyńska T., Borczak B. (2017). The Effect of Package Type on Selected Parameters of Nutritional Quality of the Chilled Stored Red Sauerkraut. J. Food Process. Preserv..

[23] Da Silva W.P., Mata M.E.R.M.C., E Silva C.D.P.S., Guedes M.A., Lima A.G.B. (2008). Determinação da difusividade e da energia de ativação para feijão macassar (*Vigna unguiculata* (L.) Walp.), variedade sempre-verde, com base no comportamento da secagem. Eng. Agrícola.

[24] da Silva W.P., Aires J.E.D.F., de Castro D.S., Silva C.M.D.P.D.S.E., Gomes J.P. (2014). Numerical description of guava osmotic dehydration including shrinkage and variable effective mass diffusivity. LWT-Food Sci. Technol..

[25] da Silva W.P., e Silva C.M., Lins M.A., Gomes J.P. (2013). Osmotic dehydration of pineapple (*Ananas comosus*) pieces in cubical shape described by diffusion models. LWT-Food Sci. Technol..

[26] Da Silva W.P., E Silva C.M., Lins M.A. (2011). Determination of expressions for the thermal diffusivity of canned foodstuffs by the inverse method and numerical simulations of heat penetration. Int. J. Food Sci. Technol..

[27] da Silva W.P., e Silva C.M.D.P.S., Farias V.S.D.O., e Silva D.D.P.S. (2010). Calculation of the convective heat transfer coefficient and cooling kinetics of an individual fig fruit. Heat Mass Transf..

[28] Mendonça S.L., Filho C.R., da Silva Z. (2005). Transient conduction in spherical fruits: Method to estimate the thermal conductivity and volumetric thermal capacity. J. Food Eng..

[29] Baïri A., Laraqi N., de María J.G. (2007). Determination of thermal diffusivity of foods using 1D Fourier cylindrical solution. J. Food Eng..

[30] Bock V., Nilsson O., Blumm J., Fricke J. (1995). Thermal properties of carbon aerogels. J. Non-Cryst. Solids.

[31] Murakami E.G. (1994). Thermal Processing Affects Properties of Commercial Shrimp and Scallops. J. Food Sci..

[32] Murakami E.G. (1997). The thermal properties of potatoes and carrots as affected by thermal processing. J. Food Process. Eng..

[33] Kumcuoglu S., Tavman S. (2007). Thermal diffusivity determination of pizza and puff pastry doughs at freezing temperatures. J. Food Process. Preserv..

[34] Monde M., Mitsutake Y. (2001). A new estimation method of thermal diffusivity using analytical inverse solution for one-dimensional heat conduction. Int. J. Heat Mass Transf..

[35] Markowski M., Bialobrzewski I., Cierach M., Paulo A. (2004). Determination of thermal diffusivity of Lyoner type sausages during water bath cooking and cooling. J. Food Eng..

[36] Ukrainczyk N. (2009). Thermal diffusivity estimation using numerical inverse solution for 1D heat conduction. Int. J. Heat Mass Transf..

[37] Muramatsu Y., Greiby I., Mishra D.K., Dolan K.D. (2017). Rapid Inverse Method to Measure Thermal Diffusivity of Low-Moisture Foods. J. Food Sci..

[38] da Silva W.P., de Medeiros M.S., Gomes J.P., e Silva C.M.D.P.S. (2020). Improvement of methodology for determining local thermal diffusivity and heating time of green coconut pulp during its pasteurization. J. Food Eng..

[39] Fan T.-H., Pang H.-Q., Zhong W.-R., Zhang S.-N., Wu X. (2022). Experiment and Inverse Analysis to Estimate SiO_2_ Aerogel Composite’s Thermophysical Properties by the Surface’s Temperature Response. Int. J. Thermophys..

[40] Rabie M.A., Soliman A.Z., Diaconeasa Z.S., Constantin B. (2015). Effect of Pasteurization and Shelf Life on the Physicochemical Properties of Physalis (*Physalis peruviana* L.) Juice. J. Food Process. Preserv..

[41] Saeeduddin M., Abid M., Jabbar S., Wu T., Yuan Q., Riaz A., Hu B., Zhou L., Zeng X. (2017). Nutritional, microbial and physicochemical changes in pear juice under ultrasound and commercial pasteurization during storage. J. Food Process. Preserv..

[42] Debbarma T., Thangalakshmi S., Tadakod M., Singh R., Singh A. (2021). Comparative analysis of ohmic and conventional heat-treated carrot juice. J. Food Process. Preserv..

[43] da Silva W.P., da Silva P., de Souto L.M., Junior A.F.D.S., Ferreira J.P.D.L., Gomes J.P., Queiroz A.J.D.M. (2022). Determination of constant and variable thermal diffusivity of cashew pulp during heating: Experimentation, optimizations and simulations. Case Stud. Therm. Eng..

[44] Da Silva W.P., E Silva C.M.D.P.S. (2014). Calculation of the convective heat transfer coefficient and thermal diffusivity of cucumbers using numerical simulation and the inverse method. J. Food Sci. Technol..

[45] da Silva W.P., e Silva C.M.D., de Souto L.M., Moreira I.D.S., da Silva E.C.O. (2018). Mathematical model for determining thermal properties of whole bananas with peel during the cooling process. J. Food Eng..

[46] Santos N.C., Silva W.P., Gomes J.P., Barros S.L., Silva C.M.D.P.S.E., Santiago M., Júnior A.F.S. (2022). Determination of thermal properties (and their uncertainties) during the cooling of apples (*Malus communis*). J. Food Process. Eng..

[47] da Silva W.P., e Silva C.M., Gama F.J. (2012). An improved technique for determining transport parameters in cooling processes. J. Food Eng..

[48] Erdoğdu F., Linke M., Praeger U., Geyer M., Schlüter O. (2014). Experimental determination of thermal conductivity and thermal diffusivity of whole green (unripe) and yellow (ripe) Cavendish bananas under cooling conditions. J. Food Eng..

[49] Incropera F.P., Dewitt D.P., Bergman T.L., Lavine A.S. (2006). Fundamentals of Heat and Mass Transfer.

[50] Erdoğdu F. (2008). A review on simultaneous determination of thermal diffusivity and heat transfer coefficient. J. Food Eng..

[51] Mari J., Mari M., Ferreira M., Conceição W., Andrade C. (2018). A simple method to estimate the thermal diffusivity of foods. J. Food Process. Eng..

[52] Chang S., Toledo R. (1990). Simultaneous Determination of Thermal Diffusivity and Heat Transfer Coefficient during Sterilization of Carrot Dices in a Packed Bed. J. Food Sci..

[53] Luikov A. (1968). Analytical Heat Diffusion Theory.

[54] Crank J. (1992). The Mathematics of Diffusion.

[55] Levenberg K. (1944). A method for the solution of certain non-linear problems in least squares. Q. Appl. Math..

[56] Marquardt D.W. (1963). An Algorithm for Least-Squares Estimation of Nonlinear Parameters. J. Soc. Ind. Appl. Math..

[57] Pereira J.C.A., da Silva W.P., da Silva R.C., e Silva C.M.D.P., Gomes J.P. (2022). Use of empirical and diffusion models in the description of the process of water absorption by rice. Eng. Comput..

[58] Fikiin K. (2021). Authentic form and origin of a popular predictive equation for specific heat capacity of unfrozen foods. Int. J. Refrig..

[59] Riedel L. (1969). Measurement of Thermal Diffusivity on Foodstuffs Rich in Water. Kaltetech.-Klim..

[60] Sweat V.E. (1974). Experimental values of thermal conductivity of selected fruits and vegetables. J. Food Sci..

[61] Silva W.P., E Silva C.M.D.P.S., Gomes J.P., Santos N.C., Queiroz A.J.M., De Figuiredo R.M.F. (2019). Calculation of the Thermal Properties (and Their Uncertainties) of Strawberry During Its Cooling Under Natural Convection. J. Agric. Sci..

[62] Da Silva W.P., E Silva C.M.D.P.S., De Souto L.M., Gomes J.P., Queiroz A.J.M., De Figueiredo R.M.F. (2019). Thermal properties determination of a cylindrical product during its cooling: Two-dimensional numerical model and uncertainty. Int. J. Food Prop..

[63] Silva W.P., Silva C.M.D.P.S., Nascimento P.L., Carmo J.E.F., Silva D.D.P.S. (2011). Influence of the geometry on the numerical simulation of the cooling kinetics of cucumbers. Span. J. Agric. Res..

[64] da Silva M.M., de Lima A.B., Gomes J.P., da Silva W.P., de Queiroz R.A., de Lima W.P.B. (2018). The Cooling and Freezing of Parallelepiped-Shaped Solid: Foundations and Application to Food Product. Diffus. Found..

[65] Brischetto S. (2014). Encyclopedia of Thermal Stresses.

